# Rapid tumor regression from PD-1 inhibition after anti-CD19 chimeric antigen receptor T-cell therapy in refractory diffuse large B-cell lymphoma

**DOI:** 10.1038/s41409-019-0657-3

**Published:** 2019-08-30

**Authors:** Brian T. Hill, Zachary J. Roberts, Allen Xue, John M. Rossi, Mitchell R. Smith

**Affiliations:** 1 Taussig Cancer Institute, Cleveland Clinic Foundation, Cleveland, OH USA; 2 Kite, A Gilead Company, Santa Monica, CA USA; 3 George Washington Cancer Center, Washington, DC USA

**Keywords:** Drug development, B-cell lymphoma

## To the Editor:

Axicabtagene ciloleucel (axi-cel) is an autologous anti-CD19 chimeric antigen receptor (CAR) T-cell therapy approved by the US Food and Drug Administration for adult patients with relapsed/refractory large B-cell lymphoma after ≥2 lines of systemic therapy [1, 2]. In ZUMA-1, the pivotal study of axi-cel in refractory large B-cell lymphoma, with a median of 27.1 months follow-up, the objective response rate was 83%, including 58% complete responses [3]. Grade ≥3 cytokine release syndrome (CRS) and neurologic events occurred in 11% and 32% of patients, respectively, and were generally reversible [3]. Significant clinical interest exists for exploring ways to augment the efficacy of CAR T-cell therapies. Programmed cell death-1 (PD-1) is expressed on antigen-activated CAR T cells [4]. In addition, checkpoint genes, including programmed death-ligand 1 (PD-L1), are upregulated in tumors of patients treated with axi-cel [5]. A recent case of a patient with primary mediastinal B-cell lymphoma (PMBCL) treated with anti-CD19 CAR T cells suggested that subsequent pembrolizumab treatment may enhance CAR T-cell activity [6]. However, as pembrolizumab demonstrated single-agent activity in PMBCL [7], it is unclear if this response resulted from direct CAR T-cell activity. This report describes a patient with rapidly progressing refractory diffuse large B-cell lymphoma (DLBCL), with strong PD-L1 expression, whose disease did not respond to axi-cel but who experienced rapid tumor regression after receiving subsequent anti-PD-1 therapy.

ZUMA-1 (NCT02348216) is a multicenter, phase 1/2 study of axi‑cel in patients with refractory large B-cell lymphoma [3, 8, 9]. After leukapheresis and manufacturing, patients received low-dose conditioning chemotherapy (cyclophosphamide 500 mg/m^2^ and fludarabine 30 mg/m^2^ intravenously, days −5 to −3) and a single intravenous axi-cel infusion (target dose, 2 × 10^6^ CAR T cells/kg, day 0). Response was assessed using International Working Group response criteria for malignant lymphoma [10]. CRS was graded per Lee et al. [11]. Neurologic events were graded using National Cancer Institute Common Terminology Criteria for Adverse Events, version 4.03. Immunohistochemistry was performed as described [8, 12]. Blood samples were analyzed for CAR T-cell and cytokine levels [13]. Each site’s institutional review board approved this study, which was conducted according to International Conference on Harmonisation Good Clinical Practice guidelines. All patients provided written informed consent. Data analyses were performed by Kite, a Gilead Company; authors had full access to data.

A 43-year-old male presented with refractory germinal center DLBCL by immunohistochemistry (CD10+, MUM1−, and BCL6+). At initial diagnosis, fluorescence in situ hybridization was negative for *MYC*, *BCL2*, and *BCL6* translocations. The patient was refractory to all prior therapies including first-line R-CHOP (rituximab, cyclophosphamide, doxorubicin, vincristine, and prednisone) and salvage R-ICE (rituximab, ifosfamide, carboplatin, and etoposide) and R-DHAP (rituximab, dexamethasone, cytarabine, and cisplatin). Positron emission tomography (PET) scans at screening showed active disease in the liver, spleen, and bones. A liver biopsy confirmed persistent DLBCL. Retrospective immunohistochemical analysis showed marked PD-L1 expression in the immediate pretreatment biopsy, with >90% of tumor and immune infiltrate expressing PD-L1 (Fig. S1). The H-score for CD19 expression in the biopsy was 190 (Fig. S1). Tumor sample genotyping was unsuccessful.

**Fig. 1:**
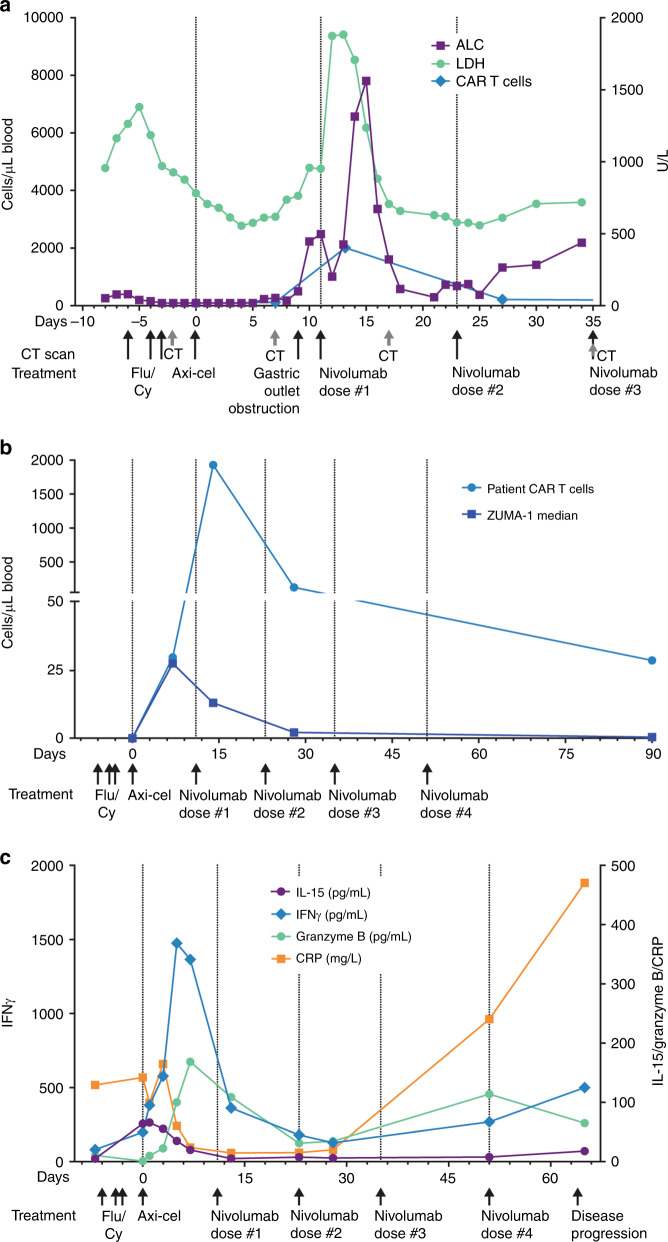
Clinical parameters and cytokine levels with axi-cel and post progression nivolumab treatment. **a** Serum LDH (right axis), ALC, and CAR T-cell levels (left axis) are shown starting at day −8, during lymphodepleting chemotherapy (Flu/Cy, days −6, −4, and −3), through axi-cel infusion (day 0) and dosing of nivolumab (days 11, 23, and 35). **b** CAR T-cell levels for the case study patient and the median for all other patients in ZUMA-1 are shown over the course of treatment. **c** Serum cytokine levels before and during treatment. ALC absolute lymphocyte count, axi-cel axicabtagene ciloleucel, CAR chimeric antigen receptor, CRP C-reactive protein, Flu/Cy fludarabine and cyclophosphamide, IFNγ interferon-γ, IL-15 interleukin-15, LDH lactate dehydrogenase, PD-L1 programmed death-ligand 1

Conditioning chemotherapy was initiated 6 days before axi-cel infusion but halted on day −5 due to fever. Fever workup including chest x-ray, blood, and urine cultures showed no evidence of infection, and fever was attributed to progressive lymphoma. Conditioning chemotherapy was administered on days −4 and −3, during which the fever abated. Abdominal computed tomography (CT) scan on day −2 confirmed multiple persistent, enlarged periportal, mesenteric, retroperitoneal, and splenic masses representing a 52% increase in index lesion volume from screening on day −43 (Fig. S2). Axi-cel (2 × 10^6^ CAR T cells/kg) was administered on day 0. By day 3, fever, tachycardia, hypoxia, and capillary leak syndrome developed, consistent with grade 3 CRS [11]. Tocilizumab 8 mg/kg (total dose 480 mg) was administered intravenously on day 3, and intravenous dexamethasone (10 mg) on day 5, with subsequent CRS resolution. However, beginning on day 1, there was clinical evidence of rapidly progressing disease, with nausea and recurrent bile duct compression associated with grade 3 direct and total hyperbilirubinemia on day 3. Abdominal pain worsened by day 5, with vomiting on day 6, and gastric outlet obstruction precluding oral intake on day 9. Serum lactate dehydrogenase (LDH) increased from 538 U/L on day 4 to 940 U/L on day 10 (Fig. 1a). A PET–CT scan on day 7 indicated progressive disease (PD; Fig. S2).

Due to PD and rapid clinical decline, the patient was moved to the long-term follow-up of ZUMA-1 and became eligible for subsequent therapy. Given its reported efficacy in DLBCL [14] and well-known safety profile, nivolumab 3 mg/kg was administered intravenously on day 11. Within 24 h, serum LDH increased twofold from 934 to 1856 U/L (Fig. 1a). The patient experienced recurrent grade 3 CRS, including grade 2 hypoxia and fever (38.1 °C) on day 15 that defervesced by day 17, and grade 1 neurologic events, followed by improvement in abdominal pain, resolution of hyperbilirubinemia, and resumed oral intake. A CT scan 6 days post nivolumab confirmed regression of abdominal lymphadenopathy and a 67% reduction in index lesion volume, consistent with partial response. Two additional doses of nivolumab were administered on days 23 and 35 after axi-cel, with no CRS or neurologic events. CT on day 35 showed continued reduction in tumor volume. However, after the fourth nivolumab infusion, the patient experienced PD on day 64. Death due to PD occurred on day 124.

To determine the contribution of CAR T cells to the unusually rapid tumor regression after nivolumab, CAR T-cell levels were examined pre and post nivolumab. Seven days post axi-cel (pre nivolumab), CAR T-cell expansion reached 30 cells/µL, equivalent to the ZUMA-1 median (26 cells/µL, *n* = 100 excluding the patient in this report). Within 2 days of nivolumab administration, CAR T-cell expansion reached a peak of 1927 CAR T cells/μL on day 13, compared with a ZUMA-1 median of 41 CAR T cells/μL (first quartile [Q1] to third quartile [Q3], 15–83; Fig. 1b). This represents a 65-fold increase above day 7 levels and ≈150-fold increase over the median day 14 level attained in ZUMA-1. Area under the curve 0 to 28 days (AUC_0–28_) from axi-cel infusion was similarly increased compared with ZUMA-1 (21,318 vs median 455 CAR T cells/μL × days [Q1–Q3, 153–920]). This rapid reexpansion was accompanied by marked peripheral lymphocytosis, with lymphocyte counts increasing from 2400 cells/dL before to 7700 cells/dL after nivolumab. CAR T-cell levels remained elevated relative to ZUMA-1 at 3 months post axi-cel (28 vs median 0.4 CAR T cells/μL). In ZUMA-1, peak CAR T expansion and AUC_0–28_ correlated with disease response, consistent with the observation made here. Biopsies of the persistent tumor masses while the patient was in response post nivolumab or of the progressive lesions at the time of progression were unavailable; thus, certain mechanisms of loss of tumor control cannot be examined. Loss of CD19 expression is known to occur in ≤50% of patients who relapse following anti-CD19 CAR T treatment. Given the apparent temporary reversal of axi-cel refractoriness with the addition of PD-1 blockade post infusion, mechanisms of CAR T exhaustion may also have contributed to loss of tumor control.

To understand the pharmacodynamic profile of CAR T-cell activity before and after nivolumab, serum cytokine levels were analyzed. Cytokines associated with proliferation (interleukin-15 [IL-15]), immune modulation (interferon-γ [IFNγ]), inflammation (C-reactive protein [CRP]), and immune effector function (granzyme B; Fig. 1c) were increased following axi-cel. While no significant secondary cytokine increase was observed, nivolumab appeared to prolong the period of elevated cytokine production, notably IFNγ and granzyme B, resulting in higher AUC_0–28_ compared with ZUMA-1 (16,315 vs 2245 pg/mL × days [Q1–Q3, 1211–4883] and 2433 vs 153 pg/mL × days [66–320], respectively). The AUC_0–28_ of IL-15 and CRP were similar to ZUMA-1 (672 vs 570 pg/mL × days [362–755] and 1754 vs 1567 mg/L × days [936–2545]).

The occurrence and resolution of clinical symptoms often associated with axi-cel post infusion and their transient recrudescence immediately following nivolumab treatment provide compelling context to the role checkpoint blockade may have played in CAR T-cell function in this patient. The increase in ferritin observed after nivolumab might indicate myeloid-related activity potentially contributing to CRS (Fig. S3). However, certain observations in this case remain difficult to interpret. The apparent discordance between the significant CAR T-cell reexpansion and the lack of obvious increases in serum analyte expression post nivolumab infusion (Fig. S3) may be related to the kinetics of changes in these analytes and the timing of blood sampling. It is possible that increased but transient expression of some immune effector molecules occurred but resolved prior to the blood draw.

This case suggests a potential role played by immune checkpoints in axi‑cel efficacy. Checkpoint blockade may work synergistically with CAR T cells. Additional work is needed to understand appropriate patient selection and timing of CAR T-cell administration relative to checkpoint blockade. The relatively short response duration observed here suggests additional mechanisms of resistance beyond PD-1/PD-L1 axis activation. A phase 1/2 study, ZUMA‑6 (NCT02926833), is prospectively evaluating checkpoint blockade after axi-cel.

## Supplementary information

Supplemental Material
