# Supplementary material for: Rapid tumor regression from PD-1 inhibition after anti-CD19 chimeric antigen receptor T-cell therapy in refractory diffuse large B-cell lymphoma
Source: Bone Marrow Transplant. 2019 Aug 30;55(6):1184–7. doi: 10.1038/s41409-019-0657-3 (PMC7269903; doi:10.1038/s41409-019-0657-3)
Supplement: Supplementary file 1 — Supplemental Material [file 41409_2019_657_MOESM1_ESM.pdf]

Supplementary Information

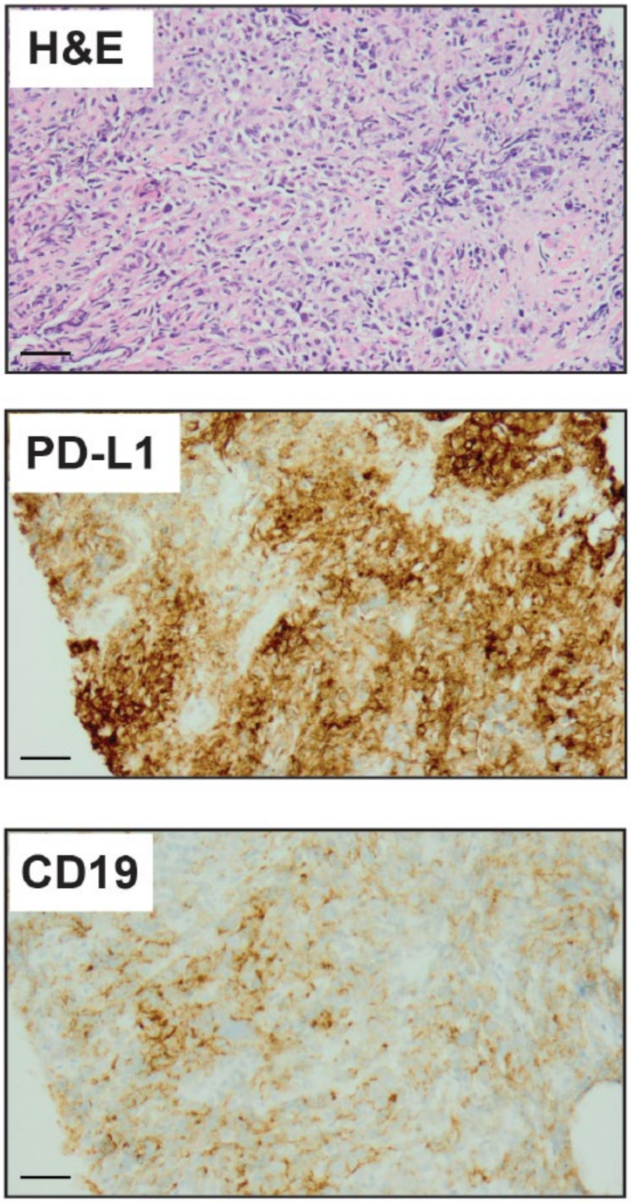

Figure S1. Retrospective analysis of tumor pretreatment PD-L1 and CD19 expression. H&E (top panel), anti-PD-L1 (middle panel), and anti-CD19 (bottom panel) immunohistochemical staining of the patient's pretreatment tumor biopsy collected on day -33. Images captured using Aperio ImageScope software (Leica Biosystems, Buffalo Grove, IL) at 40× magnification. Scale bar represents 50 μM. H&E, hematoxylin and eosin; PD-L1, programmed death-ligand 1.

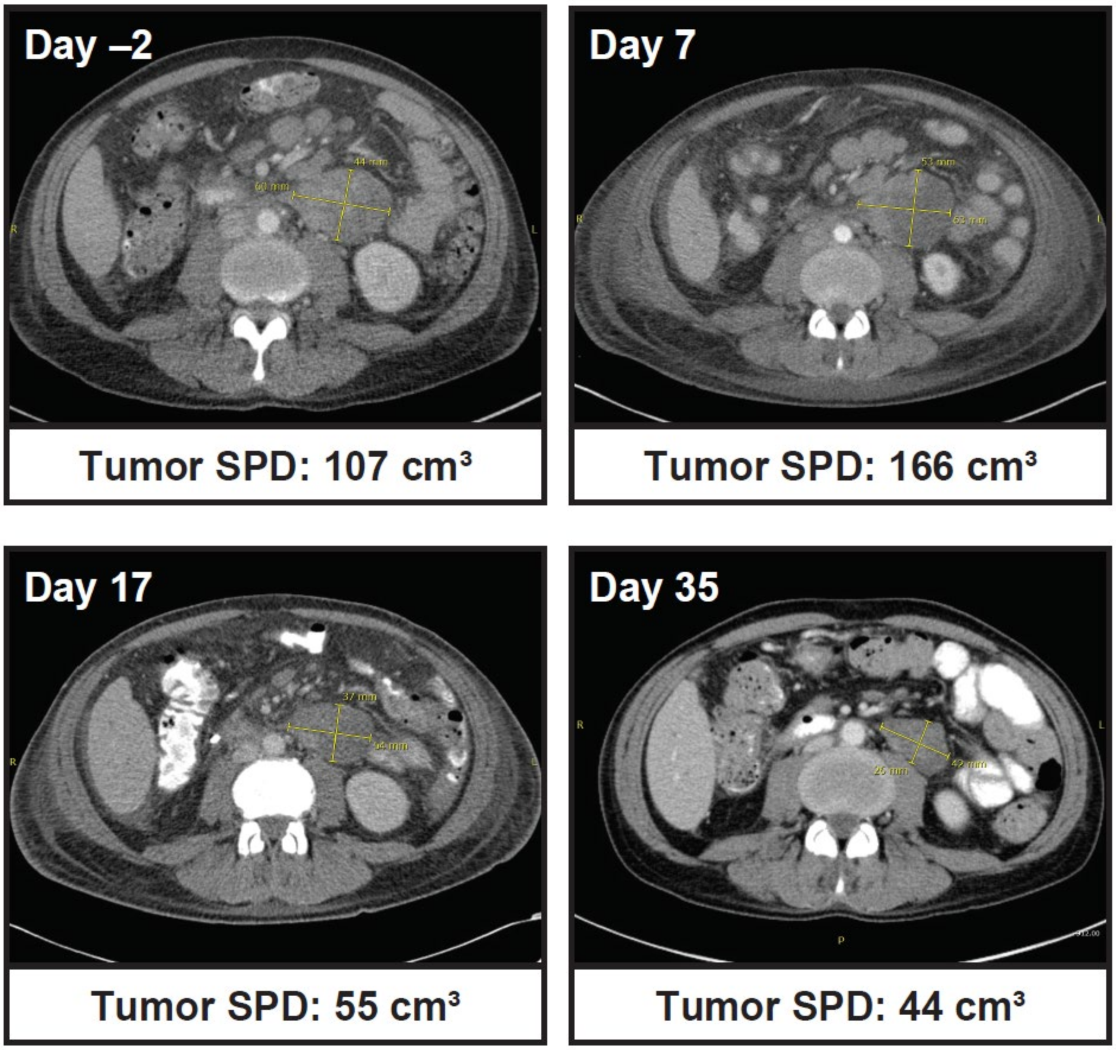

Figure S2. Representative CT scans. Serial CT images prior to axicabtagene ciloleucel infusion (day -2), at the time of clinical disease progression (day 7), 6 days after nivolumab (day 17), and 24 days after nivolumab (day 35). Tumor volume as measured by SPD of index lesions on CT scans is shown. CT, computed tomography; SPD, sum of product diameters.

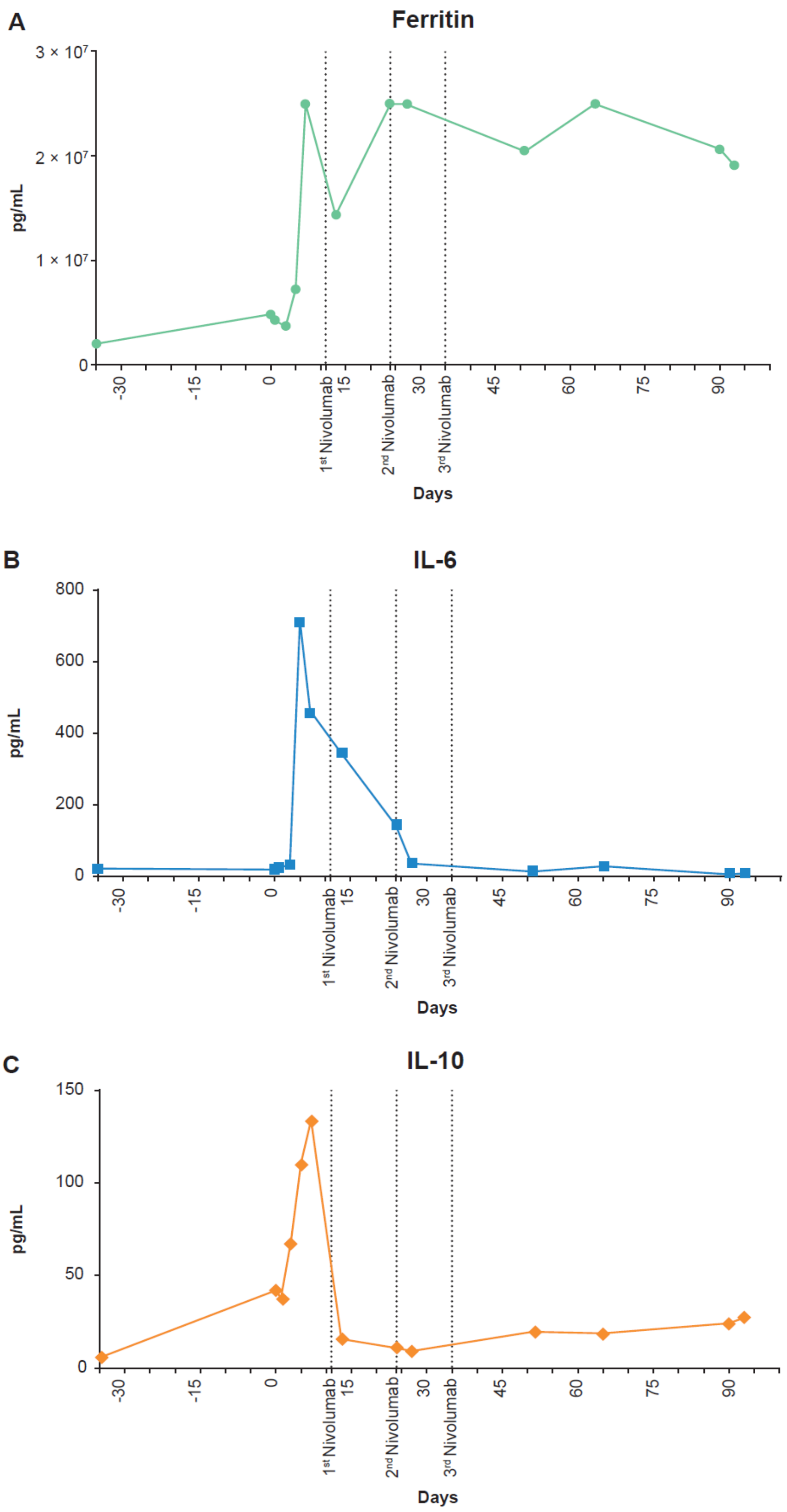
A
Ferritin
pg/mL
3 × 10^7
2 × 10^7
1 × 10^7
0
-30
-15
0
15
30
45
60
75
90
1st Nivolumab
2nd Nivolumab
3rd Nivolumab
Days
B
IL-6
pg/mL
800
600
400
200
0
-30
-15
0
15
30
45
60
75
90
1st Nivolumab
2nd Nivolumab
3rd Nivolumab
Days
C
IL-10
pg/mL
150
100
50
0
-30
-15
0
15
30
45
60
75
90
1st Nivolumab
2nd Nivolumab
3rd Nivolumab
Days

Figure S3. Cytokine levels with axi-cel and post-progression nivolumab treatment. Serum levels of (A) ferritin, (B) IL-6, and (C) IL-10 before and over the course of treatment. IL, interleukin.
